# Reverse Shoulder Arthroplasty in Patients With Rheumatoid Arthritis and Polymyalgia Rheumatica: A Clinical and Functional Review at Two Years

**DOI:** 10.7759/cureus.80191

**Published:** 2025-03-07

**Authors:** Hussain Selmi, Mohammed Tayyem, Joshua Abishek, Ali Ridha, Daoud Makki, Matt Ravenscroft

**Affiliations:** 1 Trauma and Orthopaedics, Watford General Hospital, West Hertfordshire Teaching Hospitals NHS Trust, Watford, GBR; 2 Trauma and Orthopaedics, Stepping Hill Hospital, Stockport NHS Foundation Trust, Stockport, GBR

**Keywords:** glenohumeral joint, patient-reported outcome measures, polymyalgia rheumatica, reverse shoulder arthoplasty, rheumatoid arthritis

## Abstract

Rheumatoid arthritis (RA) and polymyalgia rheumatica (PMR) are chronic inflammatory conditions that can lead to destruction and disability of the glenohumeral joint, requiring joint replacement. Disease processes can lead to further joint erosion and eventual loosening of the glenoid component of an anatomical total shoulder arthroplasty. The use of reverse shoulder arthroplasty (RSA) for RA is widely discussed; however, there is a lack of evidence relating to the use of this procedure for PMR. We conducted this study to compare outcomes of RSA in the treatment of RA and PMR.

We conducted a retrospective analysis of 30 RSA procedures, of which 18 were for RA and 12 were for PMR. All patients had significant rotator cuff damage. All patients received the same implant and the same rehabilitation protocol and were followed up at the same four intervals. At each follow-up, we assessed the range of motion and Oxford shoulder score (OSS) and undertook a radiological assessment.

We found a significant increase in the reported OSS and range of motion in both groups. We noted improvements in forward flexion, abduction and external rotation in both groups. We identified a significant increase in the OSS and post-operative range of motion after RSA in patients with RA when compared to PMR.

In conclusion, RSA remains a viable option for patients with inflammatory arthritis. However, patients with PMR show less pronounced improvement compared to those with RA.

## Introduction

Rheumatoid arthritis (RA) and polymyalgia rheumatica (PMR) are chronic inflammatory disorders involving synovial tissue that can lead to joint destruction and disability [1]. The glenohumeral joint is commonly affected in RA with a variable incidence ranging from 48% to 90% [2-5]. PMR is characterized by diffuse pain and stiffness around the shoulder and hip girdles, with synovitis being a common feature in shoulder disease [6-8]. Glenohumeral joint replacement using an unconstrained implant has generally been considered the gold-standard treatment modality to manage severe joint destruction in inflammatory arthritis [9]. Such arthroplasty procedures have been reported to provide good pain relief; however, improvement in shoulder movement and function has been less satisfactory [10,11]. The disease process in RA can lead to glenoid erosion and rotator cuff deficiency. Additionally, secondary rotator cuff failure can lead to proximal humeral migration and loosening of the glenoid component in anatomical total shoulder arthroplasty [12,13]. The results of anatomical shoulder arthroplasty have not been very promising in patients with inflammatory arthritis [11,14].

Reverse shoulder arthroplasty (RSA) was first described by Grammont in 1987 [15]. RSA can provide good functional and clinical outcomes in patients with rotator cuff arthropathy and patients with other complex shoulder disorders [16-20]. Patients with RA are immunodeficient and osteoporotic, thus increasing the risk of post-operative glenoid base plate loosening and infection [21]. In patients with PMR, the inflammation and subsequent need of corticosteroids for symptom control cause bone resorption [22]. This explains why patients with PMR may also have a high risk of glenoid component loosening and infection. Some studies have cautioned against the use of RSA in RA patients because of poor functional outcomes and high rates of complications and revision [17,23]. In contrast, some studies reported good functional outcomes when RSA was used to treat glenohumeral arthritis in patients with RA [9,24-26]. Based on our literature review, we could not find a study reporting functional and clinical outcomes following RSA in patients with PMR. Hence, we conducted this retrospective study to report clinical outcomes (range of motion, patient reported outcome measures) in patients treated with RSA for glenohumeral arthritis secondary to RA and PMR.

## Materials and methods

In our study, we included a total of 30 primary RSA procedures that were performed from March 2011 to October 2020. Patients were included if they were adults, known to have RA or PMR, underwent RSA, and had a minimum of two years of follow-up. The included patients underwent a standardised pre- and post-operative clinical evaluation and were subjected to a standardised rehabilitation protocol. Of these, 18 patients had RA, and 12 patients had PMR. All these patients had significant rotator cuff damage and/or rotator cuff compromise. The cementless Biomet (Zimmer Biomet, Warsaw, USA) comprehensive reverse shoulder prosthesis was used for all patients using a standard deltopectoral approach. If the subscapularis was intact, it was divided through the tendinous portion, approximately 1 cm medial to insertion. Repair of subscapularis tenotomy where possible was performed using non-absorbable sutures.

The post-operative rehabilitation protocol was the same for all the patients. This consisted of a simple sling for one month with immediate passive range of movements permitted up to 90 degrees of flexion and 30 degrees of external rotation.

We collected data on the clinical evaluation pre-operatively and data from follow-up at three months, six months, one year and two years. We analysed the range of active shoulder motion, Oxford shoulder score (OSS), and occurrence of any complications recorded at each interval. Furthermore, we assessed the radiographs completed at follow-up. This consisted of an anteroposterior (AP) view tangential to the base plate and lateral views looking for scapular notching, fracture, dislocation, and prosthesis loosening. Loosening of the implant was considered when either the baseplate or the stem of the humerus becomes displaced. The scapular notch was defined as the development of defects on the inferior part of the glenoid bone [27].

The pre-operative and post-operative OSS and shoulder range of motion were compared using the Wilcoxon signed-rank in the total group, while Mann-Whitney U tests were used for comparing between the PMR and RA groups. The P-value was set at <0.05.

## Results

Thirty patients underwent RSA for glenohumeral arthritis with significant rotator cuff tear or compromise and severe arthritic changes. Among these, 18 patients were known to have RA, while 12 patients had PMR. The mean age of the patients was 71.8 years (51-88). The mean OSS improved from 8.7 pre-op to 31.8 at two years of follow-up. Forward flexion improved from 48.7° to 103.6°, abduction from 49.2° +/- 19 to 104° and external rotation from 14° to 33.5° (Table 1).

**Table 1:** Functional and clinical outcomes in the total group

Total number of patients = 30	Pre-op	Two years post-op	P-value
Oxford shoulder score	8.7+/-4.4	31.8+/-7.8	<0.0001
Forward flexion (°)	48.7+/-18.9	103.6+/-18.5	<0.0001
Abduction (°)	49.2+/19.2	104+/-18.7	<0.0001
External rotation (°)	14+/-11.4	33.5+/-14.8	<0.0001

**Figure 1:**
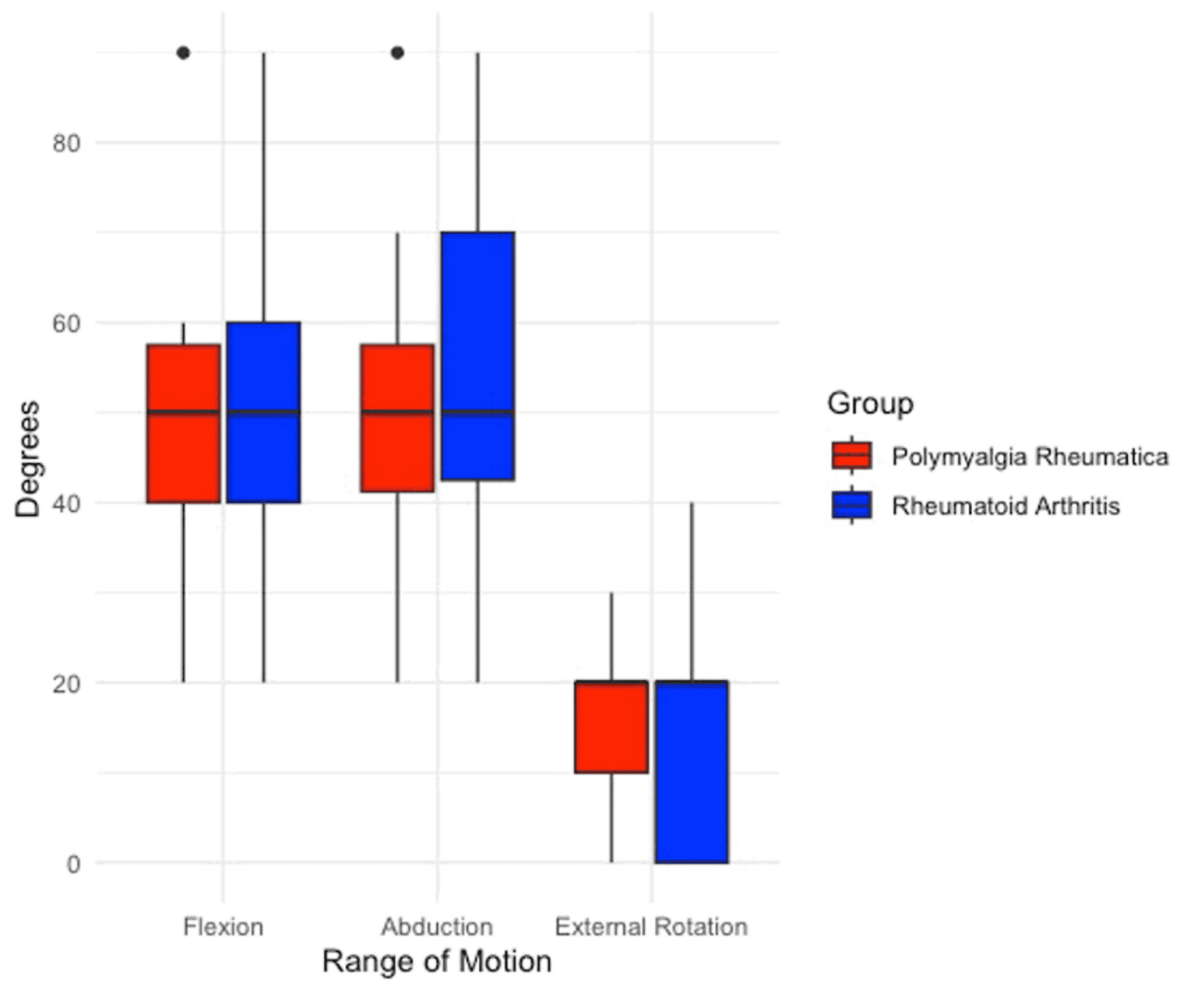
Box and whisker plots showing pre-operative range of motion assessments, comparing polymyalgia rheumatica and rheumatoid arthritis

**Figure 2:**
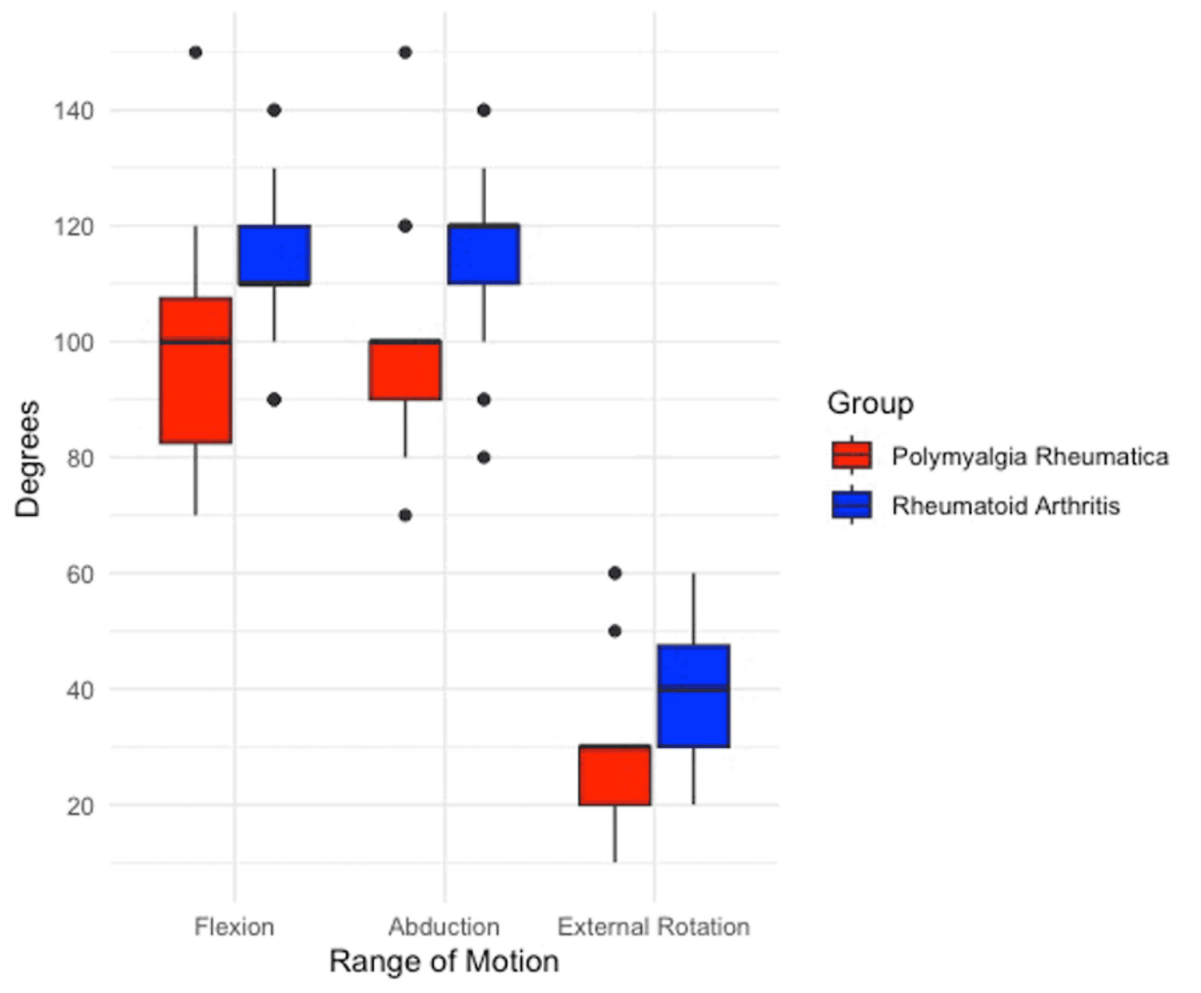
Box and whisker plots showing post-operative range of motion assessments, comparing polymyalgia rheumatica and rheumatoid arthritis

In patients with diagnosed RA, the OSS improved from 9.3 to 35.5 at a final follow-up of two years. Forward flexion improved from 56.7° to 115.8°, abduction from 57.5° to 115.8° and external rotation from 15.3° +/- 13.3 to 40.3° +/- 11.4. In patients with PMR, the OSS improved from 8.5 to 26.6 at two years follow-up. Forward flexion improved from 44.2° to 88.3°, abduction from 44.6° to 89.2°, and external rotation from 14.2° to 23.3° (Tables 2, 3 and Figures 1, 2).

**Table 2:** Patient demographics and OSS OSS: Oxford Shoulder Score

Criteria	Rheumatoid arthritis	Polymyalgia rheumatica
Number of patients	18	12
Average age (years)	69.8+/-9.7	78+/-3.9
Pre-op average OSS	9.3+/-4.5	8.5+/-4.1
Post-op average OSS (two years)	35.5+/-6.8	26.6+/-6.5

**Table 3:** Range of motion assessment ROM: Range of Motion

		Rheumatoid arthritis	Polymyalgia rheumatica
Average pre-op ROM (°)	Flexion	56.7+/-19.8	44.2+/-16.6
	Abduction	57.5+/-20.2	44.6+/-16.5
	External rotation	17.5+/-13	14.2+/-8.6
Average post-op ROM (°) - two years	Flexion	115.8+/-15	88.3+/-11.4
	Abduction	115.8+/-16	89.2+/-11.1
	External rotation	42.5+/-11.1	23.3+/-13.7

**Figure 3:**
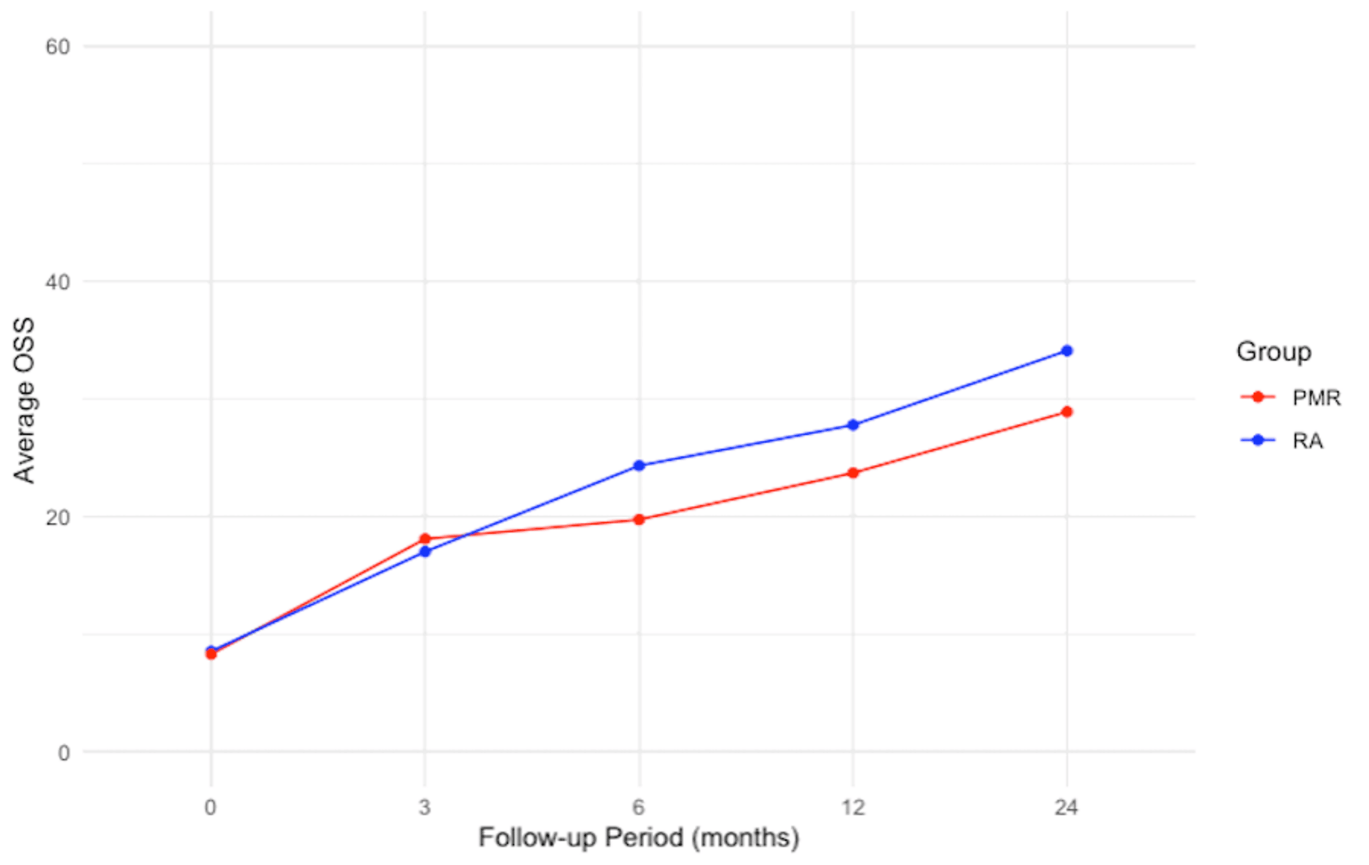
Line graph plotting oxford shoulder scores at each follow-up, comparing polymyalgia rheumatica and rheumatoid arthritis OSS: Oxford Shoulder Score; RA: Rheumatoid Arthritis; PMR: Polymyalgia Rheumatica

The improvement of the OSS and range of motion were more pronounced in the patients with RA compared to patients with PMR at two years of follow-up (Figure 3, Table 4).

**Table 4:** Post-operative comparison of statistical analysis of the polymyalgia rheumatica group and rheumatoid arthritis group

Postoperative statistical comparison	P-value
Post-operative Oxford shoulder score	0.008
Post-operative forward flexion	0.014
Post-operative abduction	0.044
Post-operative external rotation	0.002

During the follow-up period, none of our patients developed post-operative infection, fracture, or dislocation or required revision surgery. At the final radiological assessment at two years, there was no evidence of scapular notching or loosening in any patient.

## Discussion

This study aimed to evaluate the outcomes of RSA in patients with inflammatory arthritis, specifically comparing RA and PMR. To the best of our knowledge, while several studies have examined RSA outcomes in patients with RA [10-14], none have focused on PMR, making this investigation particularly relevant.

Our findings revealed that patients with RA demonstrated greater improvement in clinical scores and functional outcomes compared to those with PMR. The PMR group exhibited less significant post-operative improvements, suggesting a differential response to RSA between these two inflammatory conditions. One potential contributing factor is the age disparity between the groups, with the PMR cohort being, on average, 10 years older than the RA cohort. This aligns with existing literature indicating that RA patients often require shoulder arthroplasty at a younger age [13] due to the nature of joint destruction, which may also explain their relatively superior post-operative outcomes compared to the PMR patients.

Interestingly, despite previous concerns that RSA in inflammatory arthritis might be associated with higher complication rates due to poor bone quality and soft tissue integrity [17,23], our study did not observe such trends. We did not report any cases of post-operative complications, with no incidences of scapular notching or loosening at two years of follow-up. This contrasts with earlier studies, which suggested a higher complication burden in this population [17,23], highlighting the potential improvements in surgical techniques and implant designs in addition to biological treatment for RA.

However, several limitations of this study must be acknowledged. This is a retrospective, single-center study with a limited sample size, which may affect the generalisability of the results. Despite these constraints, the study provides novel insights, particularly into the outcomes of RSA in patients with PMR, an area previously unexplored in the literature.

## Conclusions

In conclusion, this study highlights that while RSA yields positive outcomes for patients with inflammatory arthritis, the degree of improvement appears to differ between RA and PMR patients. The relatively older age of the PMR cohort may partly explain their less pronounced functional gains. Future prospective, multi-center studies with larger sample sizes are warranted to further elucidate these findings and optimise surgical strategies for this unique patient population.
